# Supplementary material for: Neutralization of Omicron BA.1, BA.2, and BA.3 SARS-CoV-2 by 3 doses of BNT162b2 vaccine
Source: Nat Commun. 2022 Jun 23;13:3602. doi: 10.1038/s41467-022-30681-1 (PMC9225806; doi:10.1038/s41467-022-30681-1)
Supplement: Supplementary file 1 — Supplementary Information [file 41467_2022_30681_MOESM1_ESM.pdf]

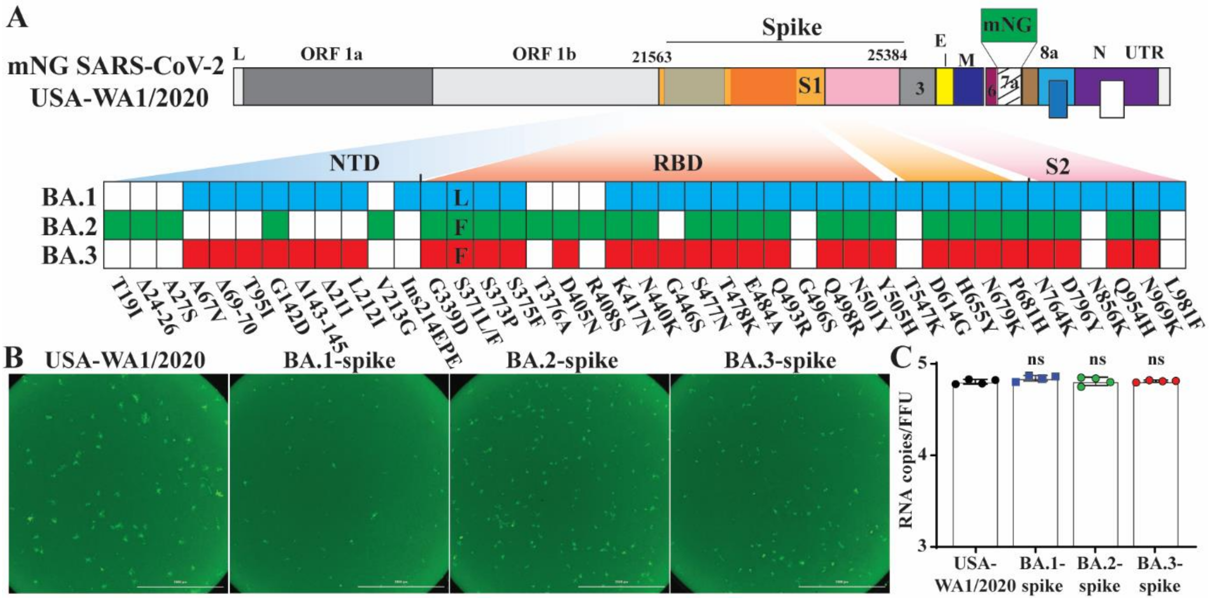

Figure S1. Construction and characterization of Omicron subvariant BA.1-, BA.2-, and BA.3-spike mNG SARS-CoV-2s. (A) Omicron BA.1-, BA.2-, and BA.3-spike mNG SARS-CoV-2s. mNG USA-WA1/2020 was used to engineer Omicron subvariant spike SARS-CoV-2s. The mNG reporter gene was engineered at the open-reading-frame-7 (ORF7) of the USA-WA1/2020 genome. Amino acid mutations, deletions (Δ), and insertions (Ins) are indicated for BA.1, BA.2, and BA.3 spikes in comparison with the USA-WA1/2020 spike. L: leader sequence; ORF: open reading frame; NTD: N-terminal domain of S1; RBD: receptor binding domain of S1; S: spike glycoprotein; S1: N-terminal furin cleavage fragment of S; S2: C-terminal furin cleavage fragment of S; E: envelope protein; M: membrane protein; N: nucleoprotein; UTR: untranslated region. (B) Fluorescent foci formed by mNG USA-WA1/2020, BA.1-, BA.2-, and BA.3-spike mNG SARS-CoV-2s. The fluorescence images were taken at 16 h after Vero E6 cells were infected with the indicated viruses in 96-well plates. Representative images from at least three independent experiments are shown. (C) Specific infectivity. The specific infectivity of mNG USA-WA1/2020 or Omicron subvariant spike mNG SARS-CoV-2 was determined by the ratio of viral genomic RNA versus fluorescent focus-forming unit ratios (genomes/FFU). Individual recombinant virus P1 stocks were measured for their viral RNA and FFUs by RT-qPCR and fluorescent focus assay on Vero E6 cells, respectively. Dots represent individual biological replicates of 4 different aliquots of viruses. Data are presented as the means ± SD. A non-parametric Mann-Whitney test (two-tailed) was used to determine the significant difference between USA-WA1/2020 and Omicron spike mNG SARS-CoV-2s. n.s., no statistical difference.

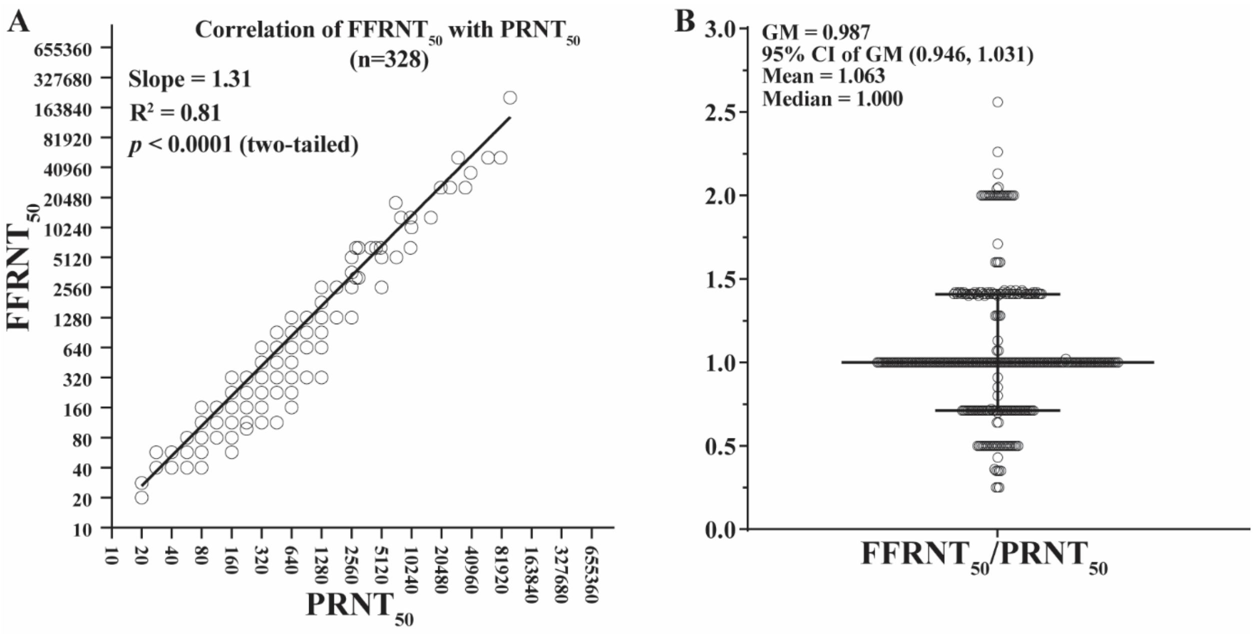

Figure S2. Correlation of neutralization titers measured by fluorescent focus-reduction neutralization test (FFRNT) and plaque reduction neutralization test (PRNT). A historical data set of BNT162b2-vaccinated sera are presented with a total of 328 tests. The neutralization titers of a serum panel were measured using mNG SARS-CoV-2-based FFRNT and conventional SARS-CoV-2-based PRNT methods. The correlation between $FFRNT_{50}$ and $PRNT_{50}$ is plotted in (A). A simple linear regression model was used to calculate the coefficiency of correlation (R^2) and the two-tailed p value. The $FFRNT_{50}/PRNT_{50}$ ratios derived from 328 human serum samples are presented in (B). GM, geometric mean; CI, confidence interval. The error bar shows the 95% CI of GM.

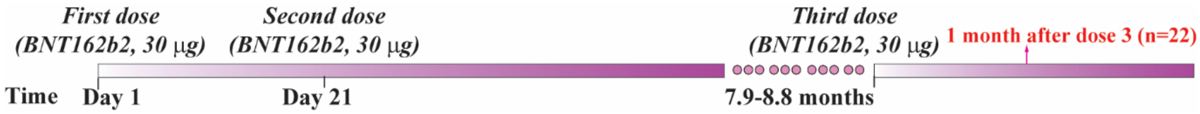

Figure S3. BNT162b2-vaccinated sera. A panel of 22 human sera was collected one month post 3 doses of the BNT162b2 vaccine. The time intervals between the three doses are indicated. This panel of sera was as recently reported.[1]

Table S1. $FFRNT_{50}$ values of BNT162b2-vaccinated sera

Subject ID	Age (Years)	Sex (F/M)	*$FFRNT_{50}$					
			USA-WA1/2022			BA.1-spike	BA.2-spike	BA.3-spike
			[&]Exp1	[&]Exp2	GMT			
1	26	F	640	905	761	320	320	226
2	28	M	1280	1810	1522	320	320	113
3	35	F	1280	1280	1280	640	453	320
4	35	F	1280	1280	1280	320	226	226
5	38	F	1280	1810	1522	640	640	320
6	38	F	320	320	320	160	160	80
7	44	F	640	453	538	113	113	80
8	44	F	2560	1280	1810	640	640	320
9	52	F	2560	1280	1810	320	320	320
10	54	M	1280	1280	1280	453	320	226
11	65	M	1280	640	905	226	226	113
12	65	M	5120	2560	3620	640	1280	320
13	66	F	640	640	640	160	160	80
14	67	M	640	905	761	320	160	113
15	68	M	640	905	761	160	113	113
16	68	F	5120	2560	3620	905	640	640
17	68	F	640	640	640	160	113	80
18	69	F	1280	905	1076	320	226	160
19	69	F	2560	1810	2153	905	640	640
20	70	M	10240	10240	10240	1280	1810	1810
21	73	M	320	453	381	40	80	28
22	74	F	1280	1280	1280	640	320	160
[†]GMT	--	--	1280	1146	1211	336	300	190
[^]95% CI	--	--	866-1893	827-1589	854-1718	234-482	210-431	127-284

*Individual $FFRNT_{50}$ value is the geometric mean of duplicate plaque assay results.

[&]Two independent experiments were performed for mNG USA-WA1/2022 because BA.1-, BA.2-, and BA.3-spike SARS-CoV-2s were tested in two experiments.

[#]The sera were collected at 1 month after dose 3 of BNT162b2 vaccine, as recently reported.[1]

[†]Geometric mean neutralizing titers.

[^]95% confidence interval (95% CI) for the GMT.

Supplemental References

1. Falsey AR, Frenck RW, Jr., Walsh EE, et al. SARS-CoV-2 Neutralization with BNT162b2 Vaccine Dose 3. N Engl J Med 2021. DOI: 10.1056/NEJMc2113468.
